# *Caenorhabditis elegans* TBX-2 Directly Regulates Its Own Expression in a Negative Autoregulatory Loop

**DOI:** 10.1534/g3.115.018101

**Published:** 2015-04-14

**Authors:** Angenee C. Milton, Peter G. Okkema

**Affiliations:** Department of Biological Sciences, University of Illinois at Chicago, Chicago, Illinois 60607

**Keywords:** T-box, *C. elegans*, SUMOylation, negative autoregulation, NF-Y

## Abstract

T-box genes often exhibit dynamic expression patterns, and their expression levels can be crucial for normal function. Despite the importance of these genes, there is little known about T-box gene regulation. We have focused on the *Caenorhabditis elegans* gene *tbx-2* to understand how T-box gene expression is regulated, and here we demonstrate TBX-2 itself directly represses its own expression in a negative autoregulatory loop. *tbx-2* is essential for normal pharyngeal muscle development, and a *tbx-2* promoter *gfp* fusion (*Ptbx-2*::*gfp*) is transiently expressed in the pharynx during embryogenesis and in a small number of head neurons in larvae and adults. Reduced *tbx-2* function resulted in ectopic *Ptbx-2*::*gfp* expression in the seam cells and gut in larvae and adults. Mutation of potential T-box binding sites within the *tbx-2* promoter resulted in a similar pattern of ectopic *Ptbx-2*::*gfp* expression, and chromatin immunoprecipitation analyses show TBX-2 binds these sites *in vivo*. This pattern of ectopic *Ptbx-2*::*gfp* expression in *tbx-2* mutants was very similar to that observed in mutants affecting the NF-Y complex, and our results comparing *tbx-2* and *nfyb-1* single- and double mutants suggest TBX-2 and NF-Y function in a single pathway to repress the *tbx-2* promoter. The *tbx-2* promoter is the first direct target identified for TBX-2, and we used it to ask whether SUMOylation is essential for TBX-2 repression. RNAi knockdown of SUMOylation pathway components led to ectopic *Ptbx-2*::*gfp* expression in the seam cells and gut. Ectopic *Ptbx-2*::*gfp* also was observed in the syncytial hypodermis, suggesting either the *tbx-2* promoter is repressed by other SUMOylation dependent mechanisms, or that decreased SUMOylation leads to stable changes in seam cell nuclei as they fuse with the syncytial hypodermis. We suggest negative autoregulation is an important mechanism that allows precise control of *tbx-2* expression levels and may allow rapid changes in gene expression during development.

T-box proteins are an ancient and highly conserved transcription factor family found in all animals, and members of this family play crucial roles in cell fate specification and organogenesis [reviewed in (Sebe-Pedros *et al.* 2013; Takashima and Suzuki 2013)]. T-box gene expression is highly regulated, and these genes often are expressed in dynamic patterns during development. For example in mouse, the closely related *Tbx2* and *Tbx3* genes are expressed at various developmental stages in tissues as different as the allantois, heart, limbs, eye, mammary gland, and brain [reviewed in (Abrahams *et al.* 2010)]. In many cases, appropriate levels of T-box gene expression are crucial for their function, and both under- and overexpression of T-box genes can lead to defects. In the mouse, progressively decreasing Tbx5 or Tbx20 activity results in distinct changes in target gene expression and phenotypic defects (Takeuchi *et al.* 2005; Mori *et al.* 2006). Likewise, in humans, haploinsufficiency for individual T-box genes underlies a variety of congenital diseases, including DiGeorge syndrome (*TBX1*), Ulnar-mammary syndrome (*TBX3*), Holt-Oram syndrome (*TBX5*), small patella syndrome (*TBX4*), and cleft palette (*TBX22* and *TBX10*) [reviewed in (Takashima and Suzuki 2013)]. Conversely, overexpression of *TBX2* and *TBX3* is found in number of different cancers, where they are believed to inhibit cellular senescence by repressing expression of the cyclin-dependent kinase inhibitors p21^WAF^ and p19^ARF^ [reviewed in (Abrahams *et al.* 2010)].

Transcription factor gene expression frequently is controlled by autoregulatory mechanisms, and both positive and negative autoregulation has been observed in organisms as different as bacteria and humans (Alon 2007; Nevozhay *et al.* 2009). Positive autoregulation generally results in slower response times after initial gene activation with an eventual switch-like increase in gene expression, and it results in cell populations that exhibit broad or even bimodal levels of gene expression. In contrast, negative autoregulation results in rapid response times and reduces cell-to-cell variation in the level of gene expression. Both positive and negative autoregulation has been described for T-box genes in ascidians and humans, suggesting autoregulation may be a common feature of this gene family (Conlon *et al.* 1996; Takahashi *et al.* 1999; Mitani *et al.* 2001; Sun *et al.* 2004; Imai *et al.* 2006; Andreou *et al.* 2007).

We are functionally characterizing T-box factors in *Caenorhabditis elegans*, and here we focus on the Tbx2 subfamily factor TBX-2. TBX-2 is the sole *C. elegans* member of the Tbx2-subfamily, which in mammals includes Tbx2, Tbx3, Tbx4, and Tbx5, and it is most closely related to the transcriptional repressors Tbx2 and Tbx3 (Pocock *et al.* 2004). In *C. elegans*, *tbx-2* is required during embryogenesis for formation of the subset of muscles in the pharynx derived from the ABa blastomere, and *tbx-2(ok529)*−null mutants arrest shortly after hatching due to an inability to feed (Roy Chowdhuri *et al.* 2006; Smith and Mango 2007). *tbx-2* mutants also exhibit defects in cell fate specification and differentiation of the hermaphrodite specific neurons and phasmid type B neurons and in olfactory adaptations (Miyahara *et al.* 2004; Singhvi *et al.* 2008). The hypomorphic mutant *tbx-2(bx59)* contains a missense mutation affecting a conserved residue in the “dimerization domain” of the T-box, and these animals exhibit partially penetrant, temperature-sensitive larval lethality (Huber *et al.* 2013). However, many of these mutants grow to adulthood, which allows the effect of reduced TBX-2 activity to be examined in later larvae and adults.

We are interested in mechanisms regulating TBX-2 activity in *C. elegans*. We have previously shown that *tbx-2* gene transcription is directly repressed by the NF-Y transcription factor, and strong genetic interactions between mutants affecting NF-Y and *tbx-2(bx59)* indicate this repression is crucial for *tbx-2* function *in vivo* (Milton *et al.* 2013). We also have shown that TBX-2 protein interacts with enzymes that attach the small ubiquitin modifier (SUMO) to target proteins and that TBX-2 can be SUMOylated in mammalian cell assays (Roy Chowdhuri *et al.* 2006; Huber *et al.* 2013). Moreover, reducing SUMOylation phenocopies *tbx-2*−null mutants and enhances the lethality and the severity of pharyngeal defects of *tbx-2(bx59)* mutants (Huber *et al.* 2013), and we hypothesize that SUMOylation is essential for TBX-2 function *in vivo*. TBX-2 can repress gene expression in cotransfection assays in mammalian cells, and a microarray comparison of wild-type and *tbx-2(bx59) C. elegans* embryos identified approximately 1000 genes that are upregulated and approximately 200 genes down-regulated in *tbx-2(bx59)* mutants, suggesting that TBX-2 predominantly functions as a transcriptional repressor.

Here we show that TBX-2 directly represses *tbx-2* gene expression in a negative autoregulatory loop. Expression of the endogenous *tbx-2* gene and a *tbx-2* promoter::*gfp* reporter is increased in *tbx-2* mutants and *tbx-2(RNAi)* animals, and ectopic *tbx-2*::*gfp* expression was found in the gut and seam cells of the lateral hypodermis in these animals. This repression was mediated by consensus T-box binding sites within the *tbx-2* promoter, and TBX-2 binds these sites *in vivo*. To test whether SUMOylation is required for TBX-2 repression, we examined the effect of inhibiting SUMOylation on *tbx-2*::*gfp* expression. We observed ectopic *Ptbx-2*::*gfp* expression in the seam cells and gut similar to that observed in *tbx-2* mutants, but we also observed more widespread *Ptbx-2*::*gfp* expression throughout the hypodermis. These results indicate that TBX-2 targets its own promoter in a negative autoregulatory loop, and suggests that TBX-2 and perhaps other factors repress *tbx-2* expression in the hypodermis and gut through SUMO-dependent mechanisms.

## Materials and Methods

### Nematode handling, transformation, and strain construction

*C. elegans* were grown under standard conditions (Lewis and Fleming 1995). Germline transformation was performed by microinjection with the plasmid pRF4 carrying *rol-6(su1006)* (100 ng/μL) and various *gfp* reporters (10 ng/μL) (Mello and Fire 1995). The following strains were used in these studies: N2, OK0592 *cuIs23[Ptbx-2*::*gfp]* III (Milton *et al.* 2013), OK0611 *cuIs25[Ptbx-2*::*gfp] X*, OK0969 *rrf-3(pk1426) II*; *cuIs23 III* (Simmer *et al.* 2002), OK0660 *tbx-2(bx59) III* (Huber *et al.* 2013), OK0460 *tbx-2(ok529)/dpy-17(e164) unc-32(e189)* (Roy Chowdhuri *et al.* 2006), OK0970 *tbx-2(bx59)*; *cuIs25 X*, OK0856 *cuEx685[Ptbx-2^prox^*::*gfp]*, OK0857 *cuEx686[Ptb-2^prox^*::*gfp]*, OK0912 *cuEx730[Ptbx-2^dist^*::*gfp]*, OK0914 *cuEx732[Ptbx-2^dist^*::*gfp]*, OK0916 *cuEx734[Ptbx-2^prox+dist^*::*gfp]*, OK0919 *cuEx737[Ptbx-2^prox+dist^*::*gfp]*, OK0873 *tbx-2(ok529)*; *wgIs159*, OK0814 *nfyb-1(cu13) II* (Milton *et al.* 2013), OK0661 *nfyb-1(cu13) II*; *tbx-2(bx59) III*, OK0898 *nfyb-1(cu13) II*; *tbx-2(bx59) III*; *cuIs25 X*, and OK1030 *tbx-2(ok529)/dpy-17(e164) unc-32(e189) III*; *cuIs25 X*. OK1029 *nfyb-1(cu13) II*; *cuIs25 X*. Newly constructed strains containing *tbx-2(ok529)*, *tbx-2(bx59)*, or *nfyb-1(cu13)* were molecularly genotyped as previously described (Roy Chowdhuri *et al.* 2006; Huber *et al.* 2013; Milton *et al.* 2013).

### General methods for nucleic acid manipulations and plasmid construction

Standard methods were used to manipulate plasmid DNAs and oligonucleotides (Ausubel 1990), and all plasmid sequences are available from the authors. The proximal T-box site was mutated in the *Ptbx-2*::*gfp* plasmid pOK206.33 (AGGTGGCA to ATTGTGC) using the Stratagene QuikChange II kit to produce *Ptbx-2^prox^*::*gfp* plasmid pOK255.04. A 42-bp fragment (−1157 to −1116 upstream of the *tbx-2* ATG) containing the pair of distal T-box sites was deleted by inverse polymerase chain reaction (PCR) of pOK206.33 or pOK255.04 with primers PO1256 [GACTCTAGAAGTGATAAGAAGCCGCGAG] and PO1257 [GACTCTAGATGCAGCACTGAATTGATGA], digested with *Xba*I, and recircularized by ligation, to produce *Ptbx-2^dist^*::*gfp* plasmid pOK278.02 and *Ptbx-2^prox+dist^*::*gfp* plasmid pOK278.03, respectively. All plasmids were sequenced to verify the presence of the mutations.

### Identification of candidate T-box binding sites in the *tbx-2* promoter

Candidate TBX-2 binding sites were identified by scanning the *Ptbx-2* promoter with the WormBase function Annotate Sequence Motif using the GBrowse plugin MotifFinder (www.wormbase.org; gmod.org/wiki/MotifFinder.pm) with a T-box half-site position frequency matrix derived from JASPAR MA0009.1 (jaspar.genereg.net) at a threshold of 0.85.

#### >T-box_half_site:

A [40 0 0 0 0 0 1 40 31 ]
C [ 0 0 0 0 0 2 7 0 5 ]
G [ 0 40 40 0 40 0 28 0 0 ]
T [ 0 0 0 40 0 38 4 0 4 ]

This position frequency matrix is nearly identical to those experimentally derived for T-box factors from the mouse and two echinoderm species (Badis *et al.* 2009; Cheatle Jarvela *et al.* 2014). This analysis identified the proximal and one distal T-box site at −259 and −1156, respectively (both AGGTGGCA). Subsequent analysis with the Uniprobe database (the_brain.bwh.harvard.edu/uniprobe/) with reduced threshold identified the second distal T-box site at −1135 (ATGTGTGA).

### RNA interference (RNAi) analyses

The feeding RNAi screen was performed with the use of *Escherichia coli* strains expressing dsRNA from transcription factor genes on OK0592 *cuIs23[Ptbx-2*::*gfp]* III or OK0969 *rrf-3(pk1426)* II; *cuIs23* III at 20° or 16°, respectively, as previously described (Milton *et al.* 2013). RNAi by injection of double-stranded RNA was performed as previously described (Fire *et al.* 1998). dsRNA produced by *in vitro* transcription (Ambion) of cDNA from *tbx-2* (yk112c4/pOK165.07) was injected into OK0592 *cuIs23[Ptbx-2*::*gfp]* strain at approximately 200−500 ng/μL (Roy Chowdhuri *et al.* 2006). Injected animals were transferred to fresh plates every 12 hr and F1 progeny laid 24−48 hr postinjection (20°) were scored for altered *Ptbx-2*::*gfp* expression.

### Quantification of *tbx-2* mRNA levels using real-time PCR

Synchronized N2 and *tbx-2(bx59)* L1 larvae were prepared by bleaching mixed-stage animals and allowing the isolated embryos to grow 12 hr on OP50 seeded NGM plates at 20° (Lewis and Fleming 1995). Total RNA isolation and real-time PCR was performed as described previously (Milton *et al.* 2013). Exonic primers spanning introns in *tbx-2* [PO1048 (TCAAAACGAGAAGGTGACGG); PO1072 (ATGTGTGGGTAGTGAAGCGG)] and the control mRNA *ama-1* [PO1061 (AGGCGAAGGATGTGTTGTG); PO1062 (TCACCGTGTTCTTTGGGTC)] in real-time PCR reactions. Three to four replicates were performed for each RNA sample, and three independently isolated RNA samples were assayed for each genotype. The *tbx-2* mRNA level relative to that of *ama-1* was compared in N2 and *tbx-2(bx59)* animals using the 2^-ΔΔCt^ method (Livak and Schmittgen 2001).

### Chromatin Immunoprecipitation (ChIP) and quantification

ChIP assays were performed on mixed-stage N2 and OK0873 *tbx-2(ok529)*; *wgIs159* animals as described (Mukhopadhyay *et al.* 2008). Animals were fed in liquid culture with *E. coli* HB101 at 20°, harvested by centrifugation, Dounce homogenized at room temperature in phosphate-buffered saline + 2% formaldehyde, incubated 20 min, and quenched by adding glycine to 120 mM. Chromatin was sheared to 200−500 bp by sonication (Branson Sonifier) at 30% amplitude for 8 cycles of 12-se pulses (0.9 sec on, 0.1 sec off), with cooling between cycles, and 10% of the lysate was saved for input. Preclearing of the lysate was performed twice, once with Dynabeads protein G (Invitrogen) bound to 2.5 μg of IgG preserum followed by preclearing with Dynabeads alone. Immunoprecipitation reactions containing approximately 2−3 mg of total protein were performed with 3 μg of anti-green fluorescent protein (anti-GFP; Roche 11814460001) or IgG preserum (Invitrogen 02-6502). No antibody controls were performed in parallel to experimental assays. Three independent assays were performed for each strain.

Crosslinking was reversed and the precipitated DNA was analyzed by semiquantitative PCR for 33 and 35 cycles to verify linear amplification. Primers flanking the proximal T-box site [PO1307 (CATCCATCCATGGACCATTC); PO1308 (CGTTTTGCCGCTCTATGACT)], the distal T-box sites [PO1311 (TCAGTGCTGCAAATGTGTGA); PO1312 (CATTCTCGCGGCTTCTTATC)], or *sax-2* [PO1394 (TGGATCATCAGTGTGTGCCT); PO1395 (AAATTCACGTTCGATCCTCG)] were used.

DNA was electrophoresed on 2–2.8% agarose gels and band intensities were measured using ImageJ (rsb.info.nih.gov/ij/). Gel images were inverted and background was subtracted with a rolling ball radius of 20. Enrichment of DNA was measured by averaging anti-GFP/Input or IgG/Input. Fold difference was calculated by difference of averaged GFP over IgG. Statistical significance was determined by two-tailed *t*-test with unequal variance comparing fold enrichment of the proximal or distal T-box sites to relative to *sax-2* in N2 and *tbx-2(ok529)*; *wgIs159* using Microsoft Excel.

### Microscopy

*C. elegans* were visualized using Zeiss Axioskop and AxioImager microscopes equipped for differential interference contrast and fluorescence microscopy. Images were captured using an Axiocam MRm camera and AxioVision software. Seam cells were identified by their position on the lateral surface of the animal and underlying the alae in L1 and adult animals (Altun and Hall 2009b). Hyp6 nuclei were identified by position in L1 animals (Altun and Hall 2009a).

## Results

### TBX-2 negatively regulates its own expression

**Figure 1:**
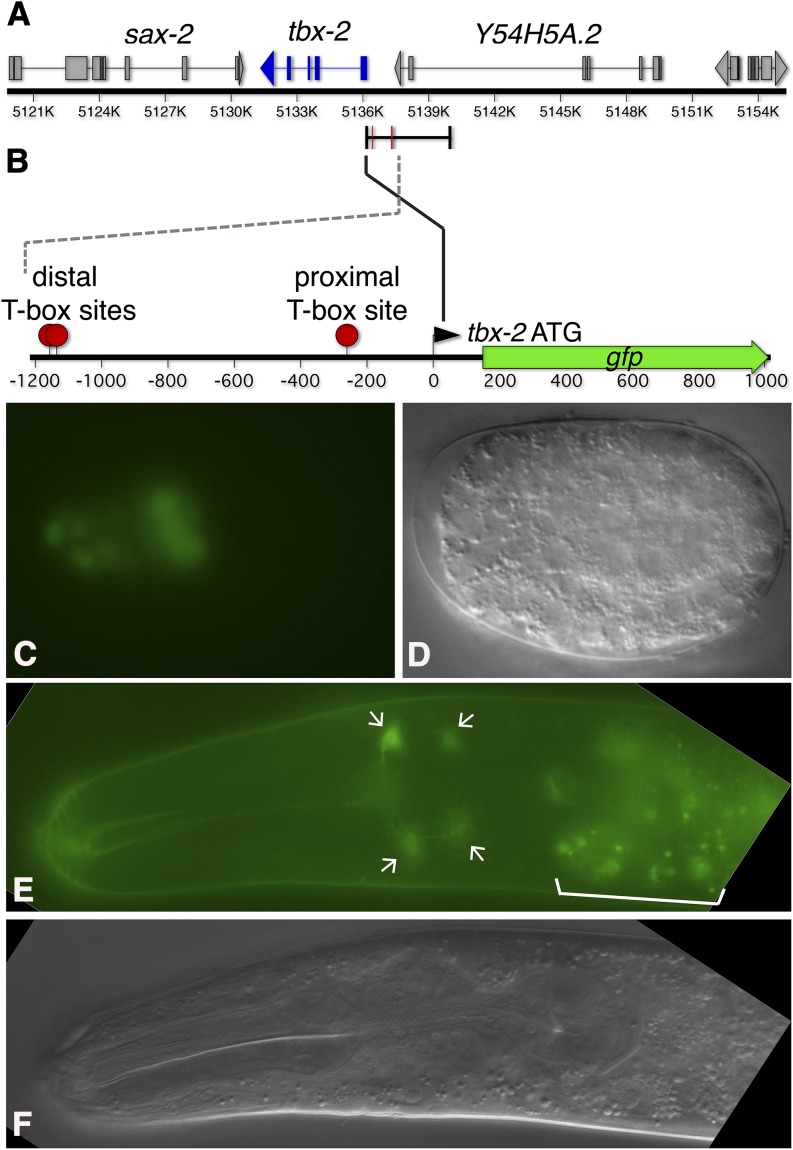
*Ptbx-2*::*gfp* expression in embryos and late larvae. (A) Schematic diagram of the *tbx-2* region contained in fosmid WRM063aG09 indicating the location of the 3.8-kb *Ptbx-2* promoter fragment and the positions of consensus T-box binding sites within this fragment (red bars). (B) Diagram of a portion of the *Ptbx-2* promoter indicating the positions of candidate T-box binding sites (red dots). For clarity the orientation of this fragment is reversed relative to (A) and only the region containing T-box sites is indicated. A promoter proximal site is located at −259 bp upstream of the *tbx*-2 ATG (AGGTGGCA, minus strand sequence), and a pair of closely spaced distal sites are located at -1135 bp (AGGTGGCA, plus strand) and −1156 bp (ATGTGTGA, plus strand) upstream of the *tbx-2* ATG, respectively. (C, D) Green fluorescent protein (GFP; left) and differential interference contrast (DIC; right) images of *Ptbx-2*::*gfp* expression in the pharyngeal primordium in an embryo at the end of gastrulation (anterior to the left). (E, F) GFP (top) and DIC (bottom) images of an L4 animal expressing *Ptbx-2*::*gfp* in head neurons (arrows). Occasional GFP expression was also observed in tail neurons, but no expression was detected elsewhere. Gut cell cytoplasm contains autofluorescent gut granules (bracket).

We identified TBX-2 as a repressor of its own promoter in an RNAi-based screen where we knocked down expression of transcription factor genes in a strain containing a *tbx-2* promoter *gfp* fusion (*Ptbx-2*::*gfp*) (Figure 1, A and B). In wild-type animals, *Ptbx-2*::*gfp* is expressed in the developing pharynx of embryos and becomes restricted to several neurons in the head near hatching, where expression persists through adulthood (Figure 1, C−F) (Roy Chowdhuri *et al.* 2006). We screened for changes in *Ptbx-2*::*gfp* expression in animals where 724 of the 924 predicted transcription factor genes were individually knocked down. Using this screen, we previously showed that knockdown of the NF-Y complex results in ectopic *Ptbx-2*::*gfp* expression in the gut and lateral hypodermal seam cells (Milton *et al.* 2013). We similarly found that *tbx-2* knockdown also results in ectopic *Ptbx-2*::*gfp* expression in these tissues. Of note, none of the other 722 transcription factor genes examined in this RNAi screen produced ectopic *Ptbx-2*::*gfp* expression, indicating that loss of TBX-2 or NF-Y activity specifically derepresses expression of this reporter.

**Figure 2:**
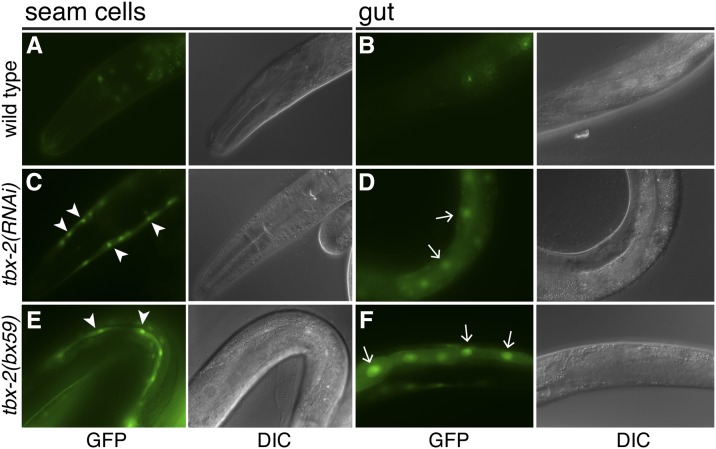
*Ptbx-2*::*gfp* is ectopically expressed in animals with reduced *tbx-2* expression. Green fluorescence protein (GFP) and differential interference contrast (DIC) images of L4 and young adult animals of the indicated genotypes expressing *Ptbx-2*::*gfp*. Ectopic *Ptbx-2*::*gfp* expression detectable in seam cells (arrowheads) and gut nuclei (arrows) are indicated in *tbx-2(RNAi)* (C, D) and *tbx-2(bx59)* mutants (E, F).

We more carefully examined *Ptbx-2*::*gfp* expression in *tbx-2(RNAi)* animals and *tbx-2* mutants. Although elimination of *tbx-2* results in severe pharyngeal defects and L1 arrest, partially affected animals grow to adulthood (Roy Chowdhuri *et al.* 2006; Huber *et al.* 2013). We found that both *tbx-2(RNAi)* animals and mutants homozygous for the temperature-sensitive hypomorphic allele *tbx-2(bx59)* exhibited highly penetrant ectopic *Ptbx-2*::*gfp* expression in the seam cells and gut in late larval and adult stages (Table 1 and Figure 2). *tbx-2(bx59)* mutants exhibited this ectopic expression at both the permissive and nonpermissive temperatures (16° and 25°, respectively), suggesting that even a relatively small reduction in TBX-2 activity derepresses the *tbx-2* promoter. This ectopic expression is specific for *Ptbx-2*::*gfp*, as we have examined expression of numerous other *gfp* reporters in *tbx-2(bx59)* mutants, including those regulated the *D2096.6*, *T25E4.1*, *pqn-71*, *myo-5*, *nmgp-1*, *T12A7.6*, *cpn-4*, and *F41H10.5* promoters, but have never observed ectopic expression in the seam cells and gut [(Huber *et al.* 2013) and data not shown].

**Table 1:** Frequency of *Ptbx-2*::*gfp* expression in seam cells and gut

Genotype	% Animals Expressing GFP in Seam Cells	% Animals Expressing GFP in Gut	n
*cuIs23[Ptbx-2*::*gfp]**^a^*	0	6	148
*cuIs23[Ptbx-2*::*gfp]*; *tbx-2(RNAi)**^a^**^,^**^b^*	97	100	228
*cuIs25[Ptbx-2*::*gfp]*; *tbx-2(bx59)**^a^* @16°	44	98	59
*cuIs25[Ptbx-2*::*gfp]*; *tbx-2(bx59)**^a^* @25°	100	100	100
*cuEx686[Ptbx-2^prox^*::*gfp]**^c^*	32	60	77
*cuEx685Ptbx-2^prox^*::*gfp* ^c^	33	53	164
*cuEx730[Ptbx-2^dist^*::*gfp]**^c^*	7	98	111
*cuEx732[Ptbx-2^dist^*::*gfp]**^c^*	2	98	178
*cuEx734Ptbx-2^prox+dist^*::*gfp**^c^*	1	96	168
cuEx737[*Ptbx-2^prox+dist^*::*gfp]**^c^*	0	99	148

GFP, green fluorescent protein.

a GFP expression was scored in L4 to young adult hermaphrodites.

b *tbx-2(RNAi)* was performed by feeding animals *E. coli* expressing *tbx-2* dsRNA.

c GFP expression was scored in L3 to young adult hermaphrodites.

**Figure 3:**
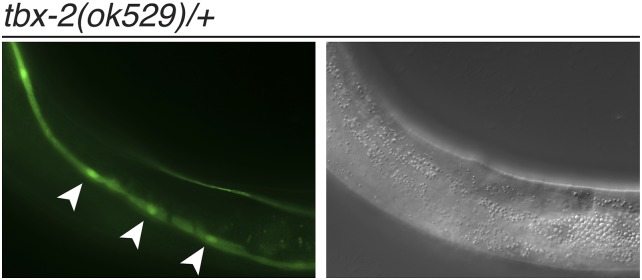
Ectopic *Ptbx-2*::*gfp* expression in *tbx-2(ok529)/+* heterozygotes. Green fluorescence protein (left) and differential interference contrast (right) images of a young adult *tbx-2(ok529)/+* heterozygote ectopically expressing *Ptbx-2*::*gfp* in seam cells (arrowheads). *tbx-2(ok529)* is a null allele containing a 1.1-kb deletion that removes sequences encoding the C-terminus of the T-box DNA-binding domain (Roy Chowdhuri *et al.* 2006).

In humans, T-box gene mutations often are haploinsufficient, with the loss of one functional allele underlying congenital diseases such as Holt-Oram and DiGeorge syndromes (Takashima and Suzuki 2013). Although *tbx-2(ok529)* null mutants are completely viable as heterozygotes (Roy Chowdhuri *et al.* 2006), we found that *tbx-2(ok529)/+* heterozygotes exhibited ectopic *Ptbx-2*::*gfp* expression in the seam cells (Figure 3). No ectopic expression was observed in the gut of these animals. Thus, *tbx-2(ok529)* also exhibits a mild haploinsufficient phenotype.

**Figure 4:**
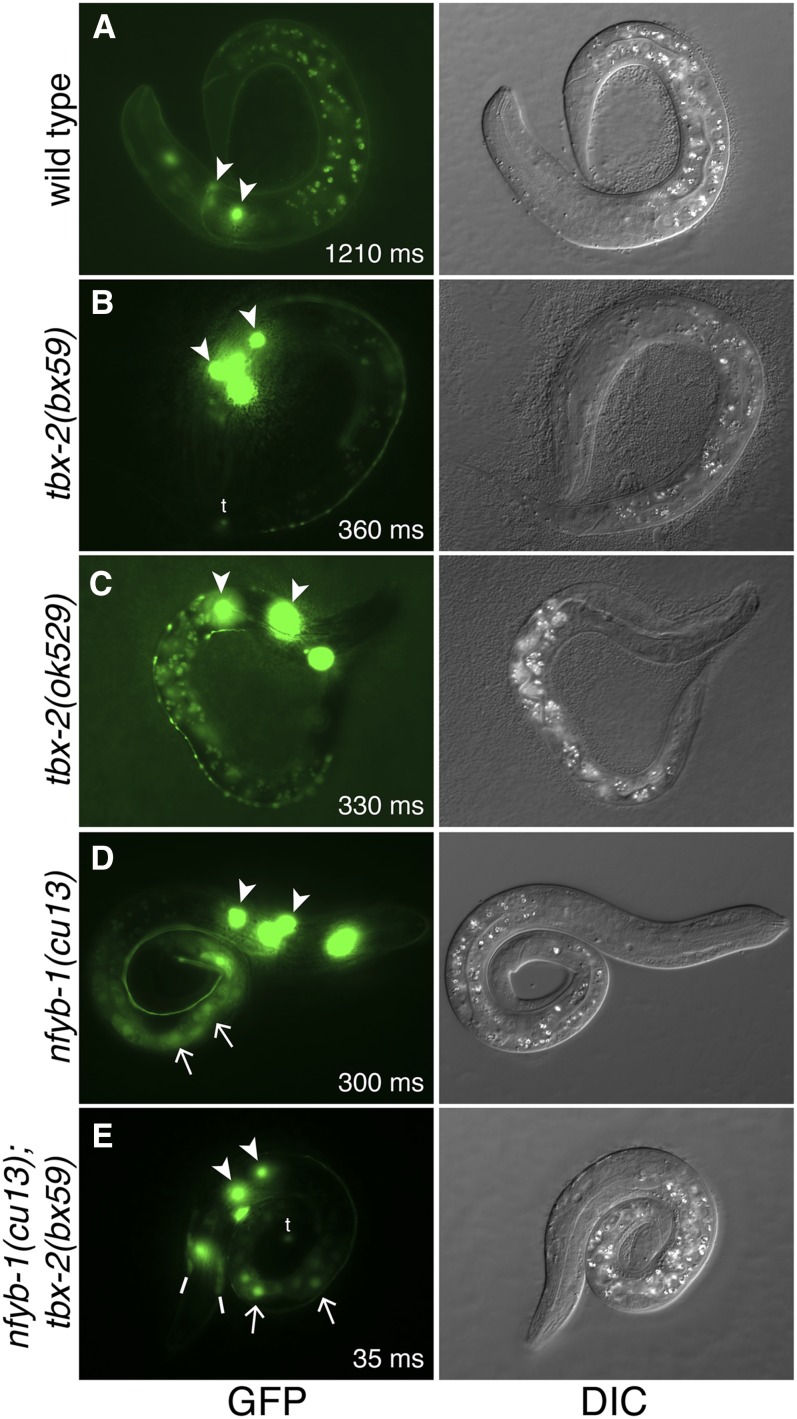
Ectopic *Ptbx-2*::*gfp* expression detectable in the gut in *tbx-2(bx59)* and *nfyb-1(cu13)*; *tbx-2(bx59)* L1 animals. Green fluorescence protein (GFP) and differential interference contrast (DIC) images of L1 animals of the indicated genotypes expressing *Ptbx-2*::*gfp*. GFP expressing head neurons (arrowheads), gut cells (arrows), hyp6 (lines), and tail neurons (t) are marked. The microscopy setup was identical for each of these images, and the exposure time for each fluorescence image is indicated in milliseconds (ms). Note that *Ptbx-2*::*gfp* is very highly expressed in head neurons in *tbx-2* and *nfyb-1* single and double mutants, and, when compared to wild-type animals, the exposure times are much shorter.

*tbx-2(ok529)* homozygotes and approximately half of *tbx-2(bx59)* animals grown at the nonpermissive temperature arrest as L1 larvae, and we examined *Ptbx-2*::*gfp* expression in these L1 animals. At this stage, no ectopic *Ptbx-2*::*gfp* was detectable in the seam cells, and only occasional expression was observed in the gut. However, these animals exhibited a strong up-regulation of *Ptbx-2*::*gfp* expression in the head neurons where *Ptbx-2*::*gfp* is normally expressed (Figure 4, A−C).

We next asked whether TBX-2 negatively regulates the endogenous *tbx-2* gene by using quantitative real-time PCR to compare *tbx-2* mRNA levels in wild-type and *tbx-2(bx59)* L1 animals grown at the nonpermissive temperature. We found *tbx-2* mRNA was overexpressed fourfold in *tbx-2(bx59)* compared with the wild type (Table 2). These results are consistent with those of a genome-wide microarray showing *tbx-2* mRNA levels are increased in *tbx-2(bx59)* mutants (Huber *et al.* 2013). Although these results using whole worm RNA cannot determine whether endogenous *tbx-2* is up-regulated in the same tissues where we observe ectopic *Ptbx-2*::*gfp* expression, they do demonstrate the endogenous gene is subject to negative autoregulation.

**Table 2:** Endogenous tbx-2 expression levels

Genotype	Relative *tbx-2* mRNA expression ± SD
+/+	1.00 ± 0.06
*tbx-2(bx59)*	4.00 ± 0.01

Collectively, these results indicate TBX-2 is a dose-dependent regulator of its own promoter. We suggest this negative autoregulation of the *tbx-2* promoter functions to precisely control *tbx-2* gene expression.

### T-box binding sites mediate *tbx-2* repression

Most T-box factors bind similar sites related to AGGTGTGA (Wilson and Conlon 2002), and we identified three potential T-box binding sites in the *Ptbx-2* promoter fragment (Figure 1A). We asked whether these sites are involved in repression of *Ptbx-2*::*gfp* and found mutation of the promoter proximal site (*Ptbx-2*::*gfp^prox^*) or deletion of a pair of closely linked distal sites (*Ptbx-2*::*gfp^dist^*) led to ectopic expression in seam cells and gut in a pattern similar to that observed when wild-type *Ptbx-2*::*gfp* expression was examined in *tbx-2* mutants and *tbx-2(RNAi)* animals (Table 1). Although these results show that both the proximal and distal sites repress *tbx-2* promoter activity, we also found that the proximal and distal sites have distinct effects on expression. Two independent transgenic lines expressed *Ptbx-2^prox^*::*gfp* at moderate frequency in both the seam cells and gut. In contrast, nearly every transgenic animal from independent lines expressed *Ptbx-2^dist^*::*gfp* in the gut, whereas relatively few of these animals expressed this construct in the seam cells. We expected that a double mutant containing both a disrupted proximal site and deleted distal sites (*Ptbx-2^prox+dist^*) would exhibit an additive expression pattern, but *Ptbx-2^prox+dist^* was expressed in a pattern very similar to the *Ptbx-2*::*gfp^dist^* single mutant, with very frequent expression in the gut but very little expression in the seam cells (Table 1). Together, these results indicate that both the proximal and distal T-box sites repress *Ptbx-2*::*gfp* expression, although their roles differ in seam cells and gut.

### TBX-2 directly represses its own promoter *in vivo*

The modENCODE Consortium provided a *tbx-2* transgene *wgIs159* containing wild-type *tbx-2* in the fosmid WRM063aG09 tagged at the 3′-end with *gfp* (Sarov *et al.* 2012). *wgIs159* expresses a full-length TBX::2GFP fusion protein, and we have found that it rescues the null mutant *tbx-2(ok529)* (P. Huber and P. Okkema, unpublished data). The modENCODE Consortium has used similar transgenes to map transcription factor binding sites throughout the genome by ChIP using anti-GFP antibodies (Niu *et al.* 2011).

**Figure 5:**
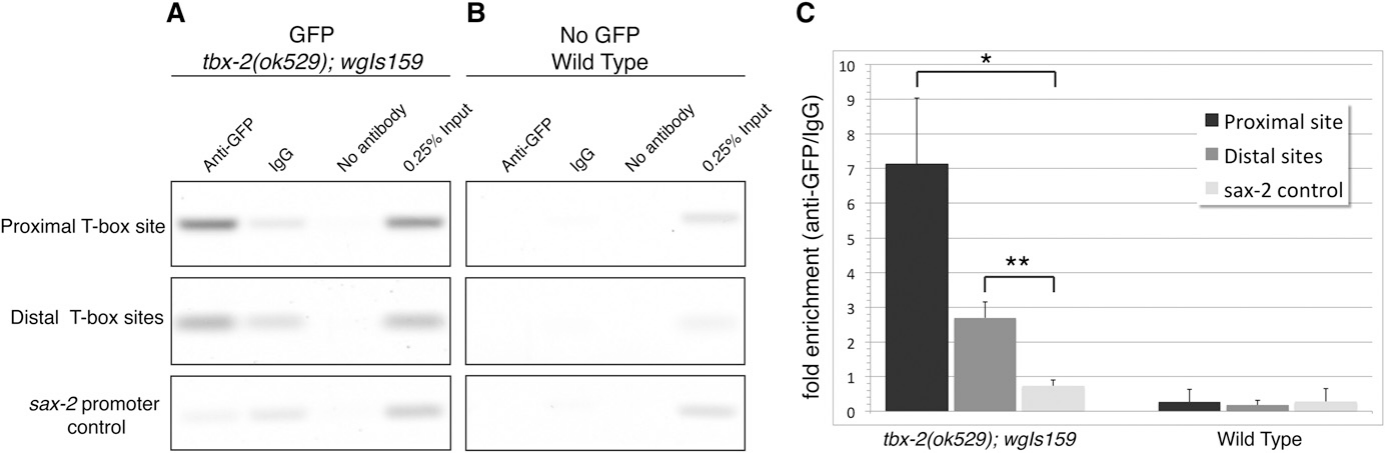
TBX-2 binds T-box sites in the *tbx-2* promoter *in vivo*. Semiquantitative polymerase chain reaction analyses of representative chromatin immunoprecipitation from *tbx-2(ok529)*; *wgIs159* animals expressing a TBX-2::GFP fusion protein (A) and wild-type N2 animals lacking any GFP (B) immunoprecipitated with anti-GFP, nonspecific IgG or no antibody as indicated. 0.25% of the total protein lysate used in the immunoprecipitation reactions is shown as input. (C) Bar graph indicating the amount of product precipitated with anti-GFP relative to the amount precipitated with IgG. Data represents the average for 3 experiments with error bars indicating standard deviation. *P* < 0.03 (*) or *P* < 0.01 (**). GFP, green fluorescent protein.

We performed ChIP on mixed-stage *tbx-2(ok529)*; *wgIs159* hermaphrodites and found TBX-2 specifically binds both the proximal and distal sites in the *tbx-2* promoter (Figure 5). Primers flanking the proximal and distal sites were used to amplify DNA isolated from chromatin immunoprecipitated with an anti-GFP antibody or with nonspecific IgG, or in mock precipitations with no antibody. Because *wgIs159* contains multiple copies of the *tbx-2* gene that increase the amount of DNA immunoprecipitated in ChIPs (Niu *et al.* 2011), we also examined binding to a region of the *sax-2* gene, which is also located in WRM063aG09 and does not contain any predicted T-box binding sites. The proximal and distal sites were enriched 7.1-fold and 2.7-fold when precipitated with anti-GFP, respectively, compared with precipitations with IgG, and this enrichment was significantly more than that observed for the *sax-2* site (Figure 5, A and C). In comparison, neither of these sites was enriched in wild-type animals lacking TBX-2::GFP protein (Figure 5, B and C). We conclude that TBX-2 specifically binds the proximal and distal T-box sites *in vivo* and that TBX-2 binding at these sites directly represses *tbx-2* expression in a negative autoregulatory loop.

### Repression *tbx-2* expression requires SUMOylation

Our previous biochemical and genetic evidence led us to hypothesize that TBX-2 function is affected by SUMOylation (Roy Chowdhuri *et al.* 2006; Huber *et al.* 2013), and this hypothesis predicts that reducing SUMOylation levels will affect *Ptbx-2*::*gfp* expression similarly to reducing TBX-2 activity. We performed RNAi to knockdown expression of the E2 SUMO conjugating enzyme UBC-9, the E3 SUMO ligase GEI-17, and the SUMO SMO-1 in a transgenic strain expressing *Ptbx-2*::*gfp*. RNAi performed by injection of *ubc-9* or *smo-1* dsRNA these genes produces highly penetrant embryonic lethality (Jones *et al.* 2002; Roy Chowdhuri *et al.* 2006) and would prevent us from examining *Ptbx-2*::*gfp* expression in larvae. Therefore, RNAi was performed by feeding animals *E. coli* expressing dsRNA to reduce the lethality of *ubc-9(RNAi)* and *smo-1(RNAi)* (Kamath and Ahringer 2003). We observed significant embryonic lethality in these experiments, indicating that the RNAi treatment was effective, but enough animals progressed through larval development to allow GFP expression to be examined in L4s and young adults.

**Figure 6:**
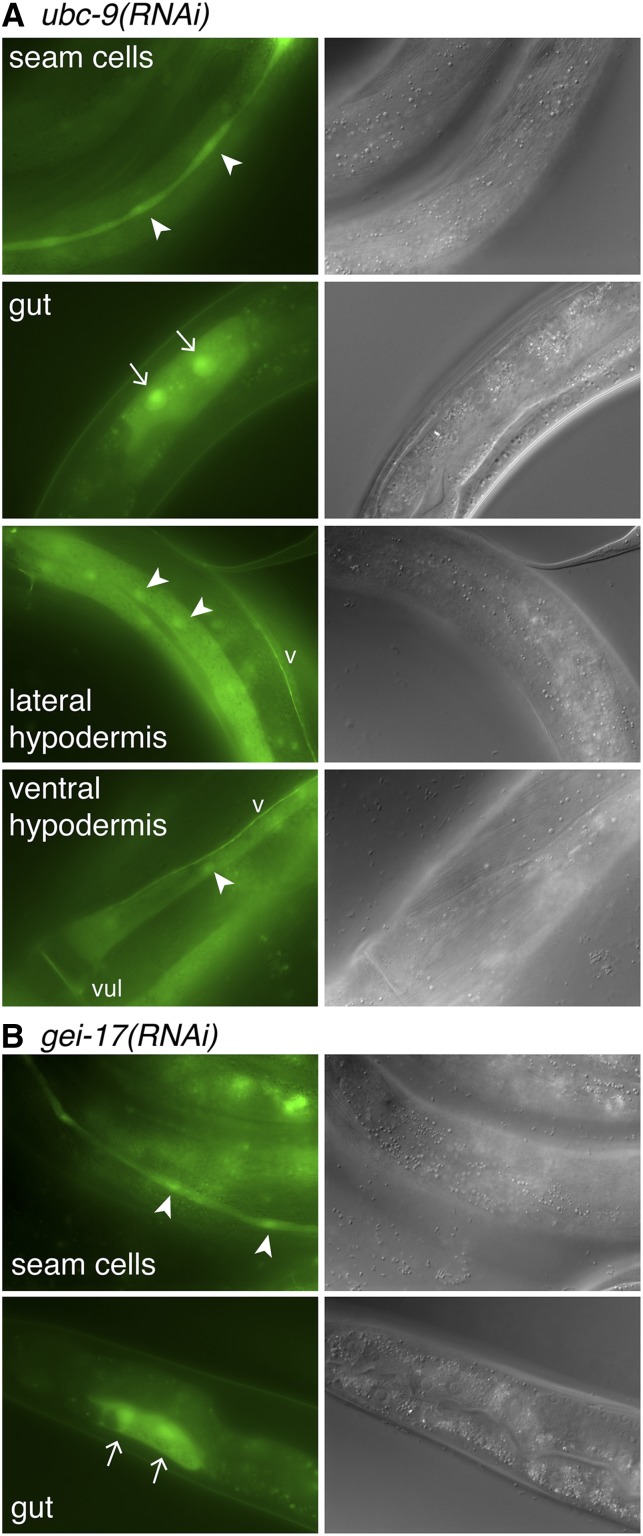
Disruption of SUMOylation causes misexpression of *Ptbx-2*::*gfp*. Green fluorescence protein (GFP; left) and differential interference contrast (right) images of young adult *ubc-9(RNAi)* (A) and *gei-17(RNAi)* (B) animals ectopically expressing *Ptbx-2*::*gfp* ectopically in the indicated tissues. Nuclei of GFP-expressing seam cells, lateral hypodermis, and ventral hypodermis are marked with arrowheads, whereas nuclei of GFP expressing gut cells are marked with arrows. ‘v’ marks the ventral nerve cord, and ‘vul’ marks the vulva on the ventral surface of the animal.

We predicted that reducing SUMOylation would lead to ectopic *Ptbx-2*::*gfp* expression in seam cells and the gut in a pattern similar to what we observed in *tbx-2(RNAi)* and *tbx-2* mutants, and we did observe *Ptbx-2*::*gfp* expression in both of these tissues in *ubc-9(RNAi)* and *gei-17(RNAi)* animals (Table 3 and Figure 6). In comparison, ectopic *Ptbx-2*::*gfp* expression was found in the seam cells of *smo-1(RNAi)* animals but was not observed in the gut. Surprisingly, we also observed frequent ectopic *Ptbx-2*::*gfp* expression in the nonseam, syncytial hypodermis in *ubc-9(RNAi)* and *smo-1(RNAi)* animals, and at a low frequency in *gei-17(RNAi)* animals (Figure 6 and Table 3). In addition, occasional *Ptbx-2*::*gfp* expression also was observed in the ventral nerve cord. Hypodermal expression typically was found in the lateral and ventral regions of the hyp7 syncytium flanking the seam cells. This pattern of ectopic *Ptbx-2*::*gfp* in the nonseam hypodermis and ventral nerve cord was only very rarely observed in *tbx-2* mutants, suggesting the *tbx-2* promoter may be repressed by additional SUMO-dependent mechanisms in these tissues. Alternatively, because the majority of hyp7 nuclei are derived from fusion with seam cell descendants (Sulston and Horvitz 1977), this ectopic expression could result from stable changes in *tbx-2* promoter activity that are maintained as seam cell nuclei join the hyp7 syncytium.

**Table 3:** Ectopic *Ptbx-2*::*gfp* expression in animals with reduced SUMOylation

Genotype*^a^*	% Animals Expressing GFP in Seam Cells	% Animals Expressing GFP in Gut	% Animals Expressing GFP in Syncytial Hypodermis	n
*Ptbx-2*::*gfp*	0	6	0	148
*Ptbx-2*::*gfp*; *tbx-2(RNAi)*	97	100	0	228
*Ptbx-2*::*gfp*; *ubc-9(RNAi)*	24	12	67	101
*Ptbx-2*::*gfp*; *gei-17(RNAi)*	40	87	6	100
*Ptbx-2*::*gfp*; *smo-1(RNAi)*	24	0	81	143

SUMO, small ubiquitin modifier; GFP, green fluorescent protein.

a Expression was scored in L4 to young adult hermaphrodites.

### *Ptbx-2*::*gfp* expression is not expanded in *nfyb-1(cu13)*; *tbx-2(bx59)* double mutants

We have previously shown that the NF-Y complex also represses *Ptbx-2*::*gfp* expression in the seam cells and gut similarly to TBX-2 (Milton *et al.* 2013). To ask whether NF-Y and TBX-2 function in parallel to repress the *tbx-2* promoter, we compared *Ptbx-2*::*gfp* expression in *nfyb-1(cu13)*; *tbx-2(bx59)* double mutants with that of wild-type animals and *tbx-2(bx59)* and *nfyb-1(cu13)* single mutants. *nfyb-1(cu13)*; *tbx-2(bx59)* double mutants exhibit temperature-sensitive, synthetic lethality at the L1 stage, so we examined expression in L1 animals grown at the nonpermissive temperature (25°).

In wild-type L1s, *Ptbx-2*::*gfp* was expressed most frequently in head neurons with less frequent expression in the anterior-most seam cell H0 and the syncytial head hypodermal cell hyp6 (Figure 4A and Table 4). Both *tbx-2(bx59)* and *nfyb-1(cu13)* single mutants exhibited a strong increase in the level of *Ptbx-2*::*gfp* expression in head neurons (Figure 4, C and D). In addition these mutants exhibited a moderately increased frequency of *Ptbx-2*::*gfp* expression in hyp6, and a small but significant increase in the number of neurons in the head and tail that expressed this construct (Table 4 and Table 5). Notably all *nfyb-1(cu13)* mutants exhibited *Ptbx-2*::*gfp* expression in H0 and the gut. The *nfyb-1(cu13)*; *tbx-2(bx59)* double mutant expressed *Ptbx-2*::*gfp* in a pattern that was very similar to the *nfyb-1(cu13)* single mutant, with very strong expression in the head neurons and frequent expression in the gut (Figure 4E and Table 4). There was also a small but significant increase in the number of *Ptbx-2*::*gfp* expressing neurons in the head and tail expression that might result from additive effects of these mutants. No additional expression was observed in other tissues in the double mutants. These results strongly suggest that NF-Y and TBX-2 do not function in independent parallel pathways to repress the *tbx-2* promoter. Furthermore, they suggest that synthetic lethality of the *nfyb-1(cu13)*; *tbx-2(bx59)* double mutant does not result from synergistic increase in *tbx-2* promoter activity.

**Table 4:** *Ptbx-2*::*gfp* ectopic expression in *tbx-2(bx59)* and *nfyb-1(cu13)* mutants at the L1 stage

Genotype	Percent L1 Animals Expressing *Ptbx-2*::*gfp* in:					n
	Head Neurons	H0 Seam Cells	Gut	Tail Neurons	Hyp6	
*cuIs23[Ptbx-2*::*gfp]*	100	14	0	0	5	56
*tbx-2(bx59)*; *cuIs25[Ptbx-2*::*gfp]*	100	38	2	73	13	60
*nfyb-1(cu13)*; *cuIs25[Ptbx-2*::*gfp]*	100	100	100	55	21	61
*nfyb-1(cu13)*; *tbx-2(bx59)*; *cuIs25[Ptbx-2*::*gfp]*	100	100	100	100	52	50

**Table 5:** Number of *Ptbx-2*::*gfp*-expressing neurons

Genotype	Head Neurons	Tail Neurons	n
*cuIs23[Ptbx-2*::*gfp]*	5.40 ± 0.7	0 ± 0	59
*tbx-2(bx59)*; *cuIs25[Ptbx-2*::*gfp]*	6.69 ± 1.7	1.23 ± 0.9	59
*nfyb-1(cu13)*; *cuIs25[Ptbx-2*::*gfp]*	7.17 ± 1.4	0.85 ± 0.9	60
*nfyb-1(cu13)*; *tbx-2(bx59)*; *cuIs25[Ptbx-2*::*gfp]*	7.76 ± 1.2	2.65 ± 1.2	47

## Discussion

Here we show that TBX-2 binds sites within its own promoter and negatively autoregulates its own expression. Knockdown of TBX-2 activity or mutation of T-box binding sites within the promoter leads to ectopic *Ptbx-2*::*gfp* expression in the hypodermal seam cells and the gut of larvae and adults and increased expression in head neurons in the L1 stage. TBX-2 protein binds these sites in the *tbx-2* promoter *in vivo*, indicating this negative autoregulation is direct. This regulation also affects endogenous *tbx-2* expression as *tbx-2* mRNA is increased in *tbx-2* mutants, although genome editing experiments could be done to verify this regulation occurs through the sites we have identified using *Ptbx-2*::*gfp* . SUMOylation is required for *tbx-2* repression, as RNAi knockdown of the UBC-9 E2 SUMO-conjugating enzyme, the GEI-17 E3 SUMO-ligase, or the SMO-1 SUMO peptide also resulted in ectopic *Ptbx-2*::*gfp* expression. These studies identify *tbx-2* as the first target regulated by TBX-2, and they demonstrate directly that TBX-2 functions as a transcriptional repressor whose function *in vivo* depends on SUMOylation. We note that using the *Ptbx-2*::*gfp* reporter as a molecular readout for TBX-2 activity facilitated our ability to detect defects in negative autoregulation, because it uncouples increased promoter activity from increased TBX-2 repressor activity.

### TBX-2 negatively autoregulates its own promoter

Negative autoregulation allows rapid transcriptional response time and reduces cell-to-cell variability in gene expression, and it can provide a linear response of target genes to the dose of key transcription factors (Alon 2007; Nevozhay *et al.* 2009). Negative autoregulation is observed for over 50% of the transcription factors in *E. coli* (Alon 2007), and it plays important roles in animal development (Crews and Pearson 2009). For example in the *Drosophila* embryo, the *Hox* factor *Ubx* maintains its expression or even completely represses its expression in different tissues through negative autoregulation (Irvine *et al.* 1993). Likewise, in the mouse and zebrafish, time delayed, negative autoregulation of basic helix-loop-helix (bHLH) transcription factors leads to oscillating gene expression levels during somitogenesis (Hirata *et al.* 2002; Lewis 2003). Negative autoregulation of transcription factors and signaling molecules is frequent in Ciona and is believed to be a key developmental feature of gene regulation (Imai *et al.* 2006).

T-box genes frequently are expressed in highly dynamic patterns, and their expression levels are critical for normal function (Naiche *et al.* 2005). Negative autoregulation may be one mechanism to precisely control T-box gene expression, and it has been observed for human *TBX22* and the Ciona T-box genes *Brachyury*, *Tbx2/3*, and *Tbx6b/c/d* (Imai *et al.* 2006; Andreou *et al.* 2007). Our studies indicate that TBX-2 negatively regulates its promoter in the head neurons, where *Ptbx-2*::*gfp* is normally expressed, and in the seam cells and gut, where *Ptbx-2*::*gfp* expression is not detected. We suggest that negative autoregulation in the neurons maintains *tbx-2* expression at levels to appropriately regulate downstream target genes. Low level expression of endogenous *tbx-2* also has been detected in the hypodermis, which includes the seam cells, and the gut (Spencer *et al.* 2011), and we suggest that negative autoregulation reversibly represses *tbx-2* expression perhaps to allow rapid *tbx-2* induction under specific developmental or environmental conditions. Although the seam and gut cells are not lineally related (Sulston *et al.* 1983), they do undergo DNA replication at each larval stage (Sulston and Horvitz 1977; Hedgecock and White 1985), and we have speculated previously that *tbx-2* may be primed for expression in these tissues to promote DNA replication (Milton *et al.* 2013).

Although knockdown of *tbx-2* results in strong derepression of *Ptbx-2*::*gfp* in both seam cells and gut, mutation of TBX-2 binding sites in the *tbx-2* promoter differently affects *Ptbx-2*::*gfp* expression in these tissues. Mutation of the proximal T-box binding site leads to ectopic expression in both the seam cells and gut, whereas deletion of the distal sites leads to frequent ectopic expression only in the gut. The double mutant containing both the proximal site mutation and deletion of the distal sites did not exhibit an additive phenotype; rather, it behaved identically to the single mutant containing the distal site deletion. These results could be explained if deletion of the distal sites not only affects TBX-2 repression but also removes a site activating expression in seam cells. We have not identified potential binding sites in this region for factors known to promote seam cell proliferation or differentiation, including GATA- and Runt-family factors (Koh and Rothman 2001; Nimmo *et al.* 2005; Smith *et al.* 2005), implying another seam cell regulatory factor might bind this deleted region.

### *tbx-2* gene repression requires SUMOylation

Our previous results led us to hypothesize that TBX-2 functions as a SUMOylation-dependent transcriptional repressor (Roy Chowdhuri *et al.* 2006; Huber *et al.* 2013). TBX-2 binds the E2 SUMO-conjugating enzyme UBC-9 and the E3 SUMO ligase GEI-17 in yeast 2-hybrid assays, and TBX-2 can be SUMOylated in mammalian cells. In *C. elegans*, RNAi knockdown of *ubc-9* phenocopies *tbx-2* null mutants, whereas *gei-17* knockdown phenocopies weak *tbx-2* mutants. Moreover, partially reducing *ubc-9* or the *smo-1* SUMO enhances the lethality and pharyngeal defects in the *tbx-2(bx59)* hypomorphic mutant, indicating SUMOylation is crucial for TBX-2 function.

Here we use the *tbx-2* promoter as a direct readout of TBX-2 repressor activity to provide additional genetic evidence that TBX-2 requires SUMOylation *in vivo*. We found that knocking down expression of *ubc-9*, *smo-1*, or *gei-17* derepresses *Ptbx-2*::*gfp* expression similarly to what we observed in *tbx-2* mutants and *tbx-2(RNAi)*. Although these results are consistent with our hypothesis, we cannot rule out the possibility that other SUMO-dependent mechanisms repress *tbx-2*. We did observe tissue specific differences in this derepression in these experiments. For example, while *tbx-2(RNAi)*, *ubc-9(RNAi)* and *gei-17(RNAi)* resulted in ectopic *Ptbx-2*::*gfp* expression in both seam cells and the gut, *smo-1(RNAi)* resulted in expression in seam cells but not in gut. This difference might result from less efficient RNAi knockdown of *smo-1* in gut than in seam cells. More interestingly, we also observed ectopic expression of *Ptbx-2*::*gfp* in the syncytial hypodermis when SUMOylation was knocked down, particularly in *ubc-9(RNAi)* or *smo-1(RNAi)* animals, but this expression was only very rarely observed in *tbx-2* mutants. This pattern of ectopic *Ptbx-2*::*gfp* expression may indicate that additional SUMO-dependent mechanisms repress expression in the syncytial hypodermis. Such mechanisms could involve transcription factors that directly repress *Ptbx-2*::*gfp* expression in the syncytial hypodermis, or mechanisms that indirectly affect *Ptbx-2*::*gfp* by affecting normal seam cell or hypodermal differentiation. These mechanisms could affect factors such as the PcG component SOP-2 or the nuclear hormone receptor NHR-25, which are SUMOylated and play important roles in seam cell and hypodermal differentiation (Zhang *et al.* 2004; Silhankova *et al.* 2005; Cai *et al.* 2008; Ward *et al.* 2013). Alternatively, as the majority of the nuclei in the syncytial hypodermis are derived from fusion with seam cell descendants (Sulston and Horvitz 1977), this ectopic expression could result from stable changes in *tbx-2* promoter activity in seam cell nuclei.

### Two mechanisms repressing *tbx-2*

We have identified the NF-Y complex and TBX-2 itself as negative regulators of *tbx-2* expression (this work; (Milton *et al.* 2013)). In both cases, these factors directly target the *tbx-2* promoter and repress *Ptbx-2*::*gfp* expression in the seam cells and gut. We did not observe increased expression of *Ptbx-2*::*gfp* expression in *nfyb-1(cu13)*; *tbx-2(bx59)* double mutants compared to each of the single mutants, strongly suggesting NF-Y and TBX-2 function together to repress *Ptbx-2*::*gfp* expression rather than through independent, parallel pathways. NF-Y has been suggested to function as a “pioneer” factor that binds its sites in chromatin, regardless of its modification state, and recruits histone modifiers to facilitate binding of other transcription factors (Fleming *et al.* 2013). In the *tbx-2* promoter, the proximal T-box site is located ∼65 bp from a functional NF-Y binding site (Milton *et al.* 2013), and we suggest NF-Y binding may facilitate TBX-2 binding to the proximal site and repressing *tbx-2* promoter activity.

*nfyb-1(cu13)*; *tbx-2(bx59)* double mutants exhibit a highly penetrant synthetic lethal phenotype indicating these factors have common functions *in vivo*, but we do not yet know the cause of this lethality (Milton *et al.* 2013). One possibility is that these factors have partially overlapping function regulating additional downstream genes, and loss of both TBX-2 and NF-Y regulation leads to double mutant lethality. This and other hypotheses can be tested by identifying and characterizing addition genes regulated by these factors.
